# Bacterial phospholipases C with dual activity: phosphatidylcholinesterase and sphingomyelinase

**DOI:** 10.1002/2211-5463.13320

**Published:** 2021-11-08

**Authors:** Laura Monturiol‐Gross, Fabian Villalta‐Romero, Marietta Flores‐Díaz, Alberto Alape‐Girón

**Affiliations:** ^1^ Instituto Clodomiro Picado Facultad de Microbiología Universidad de Costa Rica San José Costa Rica; ^2^ Centro de Investigación en Biotecnología Escuela de Biología Instituto Tecnológico de Costa Rica Cartago Costa Rica; ^3^ Departamento de Bioquímica Escuela de Medicina Universidad de Costa Rica San José Costa Rica

**Keywords:** bacterial pathogenesis, bacterial phospholipases, bacterial sphingomyelinases, bacterial toxins, virulence factors

## Abstract

Bacterial phospholipases and sphingomyelinases are lipolytic esterases that are structurally and evolutionarily heterogeneous. These enzymes play crucial roles as virulence factors in several human and animal infectious diseases. Some bacterial phospholipases C (PLCs) have both phosphatidylcholinesterase and sphingomyelinase C activities. Among them, *Listeria* *monocytogenes* PlcB, *Clostridium perfringens* PLC, and *Pseudomonas aeruginosa* PlcH are the most deeply understood. *In silico* predictions of substrates docking with these three bacterial enzymes provide evidence that they interact with different substrates at the same active site. This review discusses structural aspects, substrate specificity, and the mechanism of action of those bacterial enzymes on target cells and animal infection models to shed light on their roles in pathogenesis.

## Graphical abstract

The aim of this review is to discuss similarities and differences among the most explored bacterial enzymes that have both phospholipase C and sphingomyelinase C activities. The structural aspects of three of those enzymes, their substrate specificity, and *in silico* substrate docking analysis are discussed, as well as their mechanisms of toxicity in cultured cells and animal models.

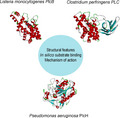

**Abbreviations**
CHO: Chinese hamster ovary
CpPLC: *Clostridium perfringens* PLC
EDTA: ethylenediaminetetraacetic acid
EGTA: egtazic acid
HUVEC: human umbilical vein endothelial
LLO: listeriolysin O
LmPlcB: *Listeria* *monocytogenes* PLC
Mpl: Listerial metalloprotease
PaPlcH: *Pseudomonas aeruginosa* PlcH
PC: phosphatidylcholine
PE: phosphatidylethanolamine
PG: phosphatidylglycerol
PI: phosphatidylinositol
PLCs: phospholipases C
PS: phosphatidylserine
ROS: reactive oxygen species
SM: sphingomyelin
TrkA: Tyr kinase A receptor

Bacterial phospholipases and sphingomyelinases are a structurally and evolutionarily heterogeneous group of lipolytic esterases [1, 2, 3]. These bacterial enzymes generate metabolites identical to the second messengers produced by eukaryotic enzymes, which play essential roles in physiological processes [1]. They are critical in the pathogenesis of various infectious diseases as they favor bacterial invasion and survival [1, 2, 3]. In some cases, they cause lysis of the host cells, thereby helping pathogens acquire some nutrients from the host, such as iron, phosphate and alternative carbon sources [1, 2, 3]. Some of those bacterial enzymes alter membrane lipid homeostasis by changing the balance of signaling molecules and thus diverting cellular processes driven by lipids to the benefit of the bacteria [1, 2, 3]. They may hydrolyze vacuolar lipids, causing phagosomal escape or phagocytosis hindrance, favoring intracellular survival, immune response evasion, and infection establishment [1, 2, 3].

Although most bacterial lipolytic esterases have either phospholipase or sphingomyelinase activity, some of them cleave glycerophospholipids and sphingomyelin (SM) [1]. Among them, *Listeria monocytogenes* PLC (LmPlcB), *Clostridium perfringens* PLC (CpPLC), and *Pseudomonas aeruginosa* PlcH (PaPlcH) are the most deeply explored. CpPLC and LmPlc are zinc metalloenzymes, whereas PaPlcH belongs to the PLC/phosphatase superfamily. This review aims to discuss the similarities and differences among those bacterial enzymes. Although the regulation of their expression is beyond the scope of this review, the structural aspects of LmPlcB, CpPLC, and PaPlcH, their substrate specificity and mechanisms of toxicity both in cultured cells and animal models are discussed below.

## The pathogenic bacteria that produce these phospholipases

*L. monocytogenes* is a Gram‐positive facultative intracellular bacterium that causes severe foodborne infections [4]. The bacterium begins its intracellular life cycle within a membrane‐bound vacuole from which it must escape to survive [3, 4]. Once into the host cytosol, *L. monocytogenes* multiplies and spreads from cell to cell using an actin‐based mechanism of motility [3, 4]. This spreading event forms a double‐membrane vacuole from which the bacterium escapes to perpetuate the intracellular life cycle [3, 4]. *L. monocytogenes* secretes several toxins that favor phagosomal escape, including LmPlcB and a phosphatidylinositol‐PLC (PI‐PLC) [3, 5]. Once bacteria have entered the bloodstream through the intestinal epithelium and lymph nodes, they can invade the liver, the spleen, and the brain [3, 4].

*C. perfringens* is a Gram‐positive anaerobic bacterium that produces more than 17 toxins [6]. This bacterium causes several diseases in humans and animals [7, 8, 9]. CpPLC, also called alpha‐toxin, is produced by all *C. perfringens* strains and is the main virulence factor in gas gangrene in humans [10]. This disease is an acute and life‐threatening soft tissue infection characterized by significant thrombosis, severe myonecrosis, and gas accumulation at the infection site [11]. Gas gangrene occurs after the introduction of the bacteria in a deep lesion or in a surgical wound [11]. CpPLC helps *C. perfringens* escape the phagosome and persist in the cytoplasm of macrophages during the earliest infection stages [11]. Then, CpPLC contributes by creating an anaerobic environment, optimal for bacterial growth, and spread [11].

*P. aeruginosa* is a Gram‐negative opportunistic pathogen associated with acute and chronic infections in predisposed human subjects [12]. *P. aeruginosa* lung infections are common and severe in chronic obstructive pulmonary disease and cystic fibrosis [12]. *P. aeruginosa* PlcH releases phosphocholine from SM and phosphatidylcholine (PC), the major components of the pulmonary surfactant, thus aiding in bacterial dispersion [12].

## Structural features comparison

LmPlcB is a 33 kDa single‐domain zinc metalloenzyme secreted as a precursor. Its activation requires the cleavage of an amino‐terminal pro‐peptide by either a listerial metalloprotease (Mpl) or host proteases, resulting in the active 29 kDa enzyme [13, 14, 15]. A His residue in Mpl plays a role in the interaction with LmPlcB for cleavage [16]. LmPlcB shares 38.7% sequence identity as with *B*. *cereus* PLC [15], which binds three Zn^+2^ ions at the active site, although LmPlcB has a considerably weaker affinity for Zn^+2^ than the *B. cereus* enzyme [14].

**Fig. 1:**
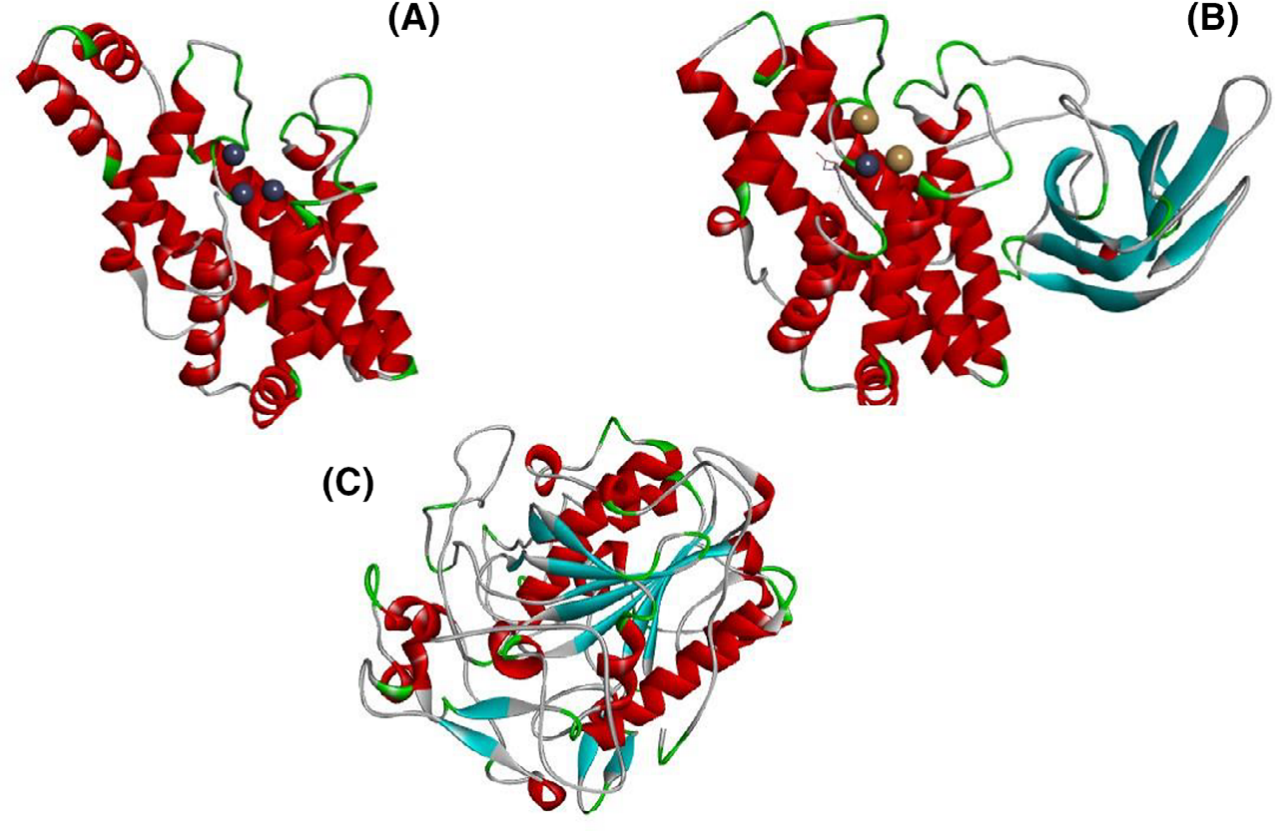
Cartoon representation of the tertiary structure of three bacterial PLCs, which play a role as virulence factors: (A) *L. monocytogenes* PlcB model built using as template *B. cereus* PLC (PDB accession No. 1ah7). (B) *C. perfringens* alpha‐toxin three‐dimensional structure (PDB accession No. 1ca1). (C) *P. aeruginosa* PaPlcH model built using as template the *Francisella tularensis* Acid Phosphatase A (PDB accession No 2d1g). The α‐helices, β‐sheets, and loops are labeled in red, cyan, and gray, respectively.

The LmPlcB amino acid sequence (Accession number Q6EAJ1) was retrieved from the UniProt database [17] (https://www.uniprot.org/) to build a structural model with SWISS‐MODEL [18] (http://swissmodel.expasy.org/) using *B. cereus* PLC (PDB accession No. 1ah7) as a template. The predicted structure shows a α‐helical single‐domain protein with three zinc atoms, as visualized using Discovery Studio Visualizer (Fig. 1A).

CpPLC is a 45 kDa zinc metalloenzyme composed of two domains joined by a short flexible linker region [19, 20]. The N‐terminal domain (residues 1–246) contains the active site and consists of nine tightly packed alpha‐helices [19]. CpPLC contains three essential Zn^+2^ atoms in its N‐terminal domain, as also observed for *B. cereus* [21] and *L. monocytogenes* PLC [13], and these could be removed by ethylenediaminetetraacetic acid (EDTA) or o‐phenanthroline. Asp56 is a critical residue in the Zn^+2^ binding site of CpPLC [22], and the D56G substitution changes the secondary structure and abolishes toxicity [23]. The C‐terminal domain (residues 256–370) consists of an eight‐stranded antiparallel beta‐sandwich required for the Ca^+2^‐dependent interaction with substrates [19, 24]. A ‘closed’ form of CpPLC has been described, with one Zn^+2^ lost and a hidden catalytically active site, and with an additional alpha helix that binds to the N‐ and C‐terminal domains [20]. An *in silico* study of CpPLC evidences at least six pairs of amino acid contacts between N‐ and C‐terminal domains [25]. The structure of *C. perfringens* alpha‐toxin (PDB accession No. 1ca1) is shown in Fig. 1B.

Structurally, CpPLC lacks two helices in the N‐terminal domain present in *B. cereus* PLC [19], resulting in a planar surface that interacts directly with the target membrane. CpPLC could can anchor the membrane by its Ca^+2^ binding sites in the C‐terminal domain and orienting its active catalytic site to interact directly with membrane phospholipids [19]. The absent *B. cereus* helix hairpin is replaced with a tryptophan residue, which, together with several hydrophobic residues in the C‐terminal domain (Phe334, Tyr 331), is well placed to interact with the hydrophobic membrane interior. In addition, the C‐terminal domain, analogous to C2 domains, binds Ca^+2^ ions but does not complete their coordination spheres, and has many positively charged residues (such as Lys300) placed to interact with the phosphate at the membrane surface [1, 19]. These adaptations for membrane interaction likely explain the higher cytotoxicity of CpPLC compared to its *B. cereus* homologue [7, 19].

PaPlcH does not share any structural similarity with LmPlcB or CpPLC, but has 23% sequence identity, as the acid phosphatase AcpA from *F. tularensis* [26, 27]. Thus, PaPlcH is the paradigm member of the PLC/phosphatase superfamily, with members in various prokaryotic species, including *M. tuberculosis*, *Bordetella* spp., *F. tularensis*, and *Burkholderia* spp, and homologs in fungi (*Aspergillus fumigatus*) and plants (*Arabidopsis*) [27]. PaPlcH is secreted via the twin‐arginine translocation and type II Xcp‐dependent systems, and it forms a multimeric complex with the chaperones PlcR1 or PlcR2 [28, 29, 30, 31].

To build a structural model of PaPlcH, its amino acid sequence (Accession number P06200) was retrieved from the UniProt database (https://www.uniprot.org/) [17]. The PaPlcH three‐dimensional structure was modeled with SWISS‐MODEL using the *F. tularensis* Acid Phosphatase A (PDB accession No 2d1g) as a template [18] (http://swissmodel.expasy.org/). The predicted structure (Fig. 1C) was visualized using Discovery Studio Visualizer.

## Substrate specificity comparison

LmPlcB hydrolyzes PC, SM, phosphatidylethanolamine (PE), phosphatidylserine (PS), PI, cardiolipin, phosphatidylglycerol (PG), plasmalogens, and plasmenylethanolamine [13, 32]. The hydrolysis mechanism of PI by LmPlcB differs from that of *L. monocytogenes* PI‐PLC because it generates a different product [32]. Accordingly, recombinant LmPlcB hydrolyses a range of phospholipids with different head groups [14]. On large unilamellar vesicles, LmPlcB has the highest activity on PE, PS‐rich mixtures, which are expected to be the main phospholipids on the inner membrane of the double‐membrane vacuole that is in contact with *L. monocytogenes* during cell‐to‐cell spread [33]. Its enzymatic activity has been widely related to disrupting the primary vacuole in certain cell types [34, 35, 36, 37, 38] and spreading vacuoles in others [39, 40]. The dual enzymatic activity of LmPlcB allows membrane fusion, which could be important for *L. monocytogenes* cell‐to‐cell spreading [33]. Interestingly, phosphocholine, a product of LmPLC enzymatic activity on PC, is a potent inhibitor of Listeriolysin O (LLO), a pore‐forming toxin secreted by *L. monocytogenes* that has cytotoxic effects on host cells during infection, thus promoting bacterial intracellular survival [41].

**Fig. 2:**
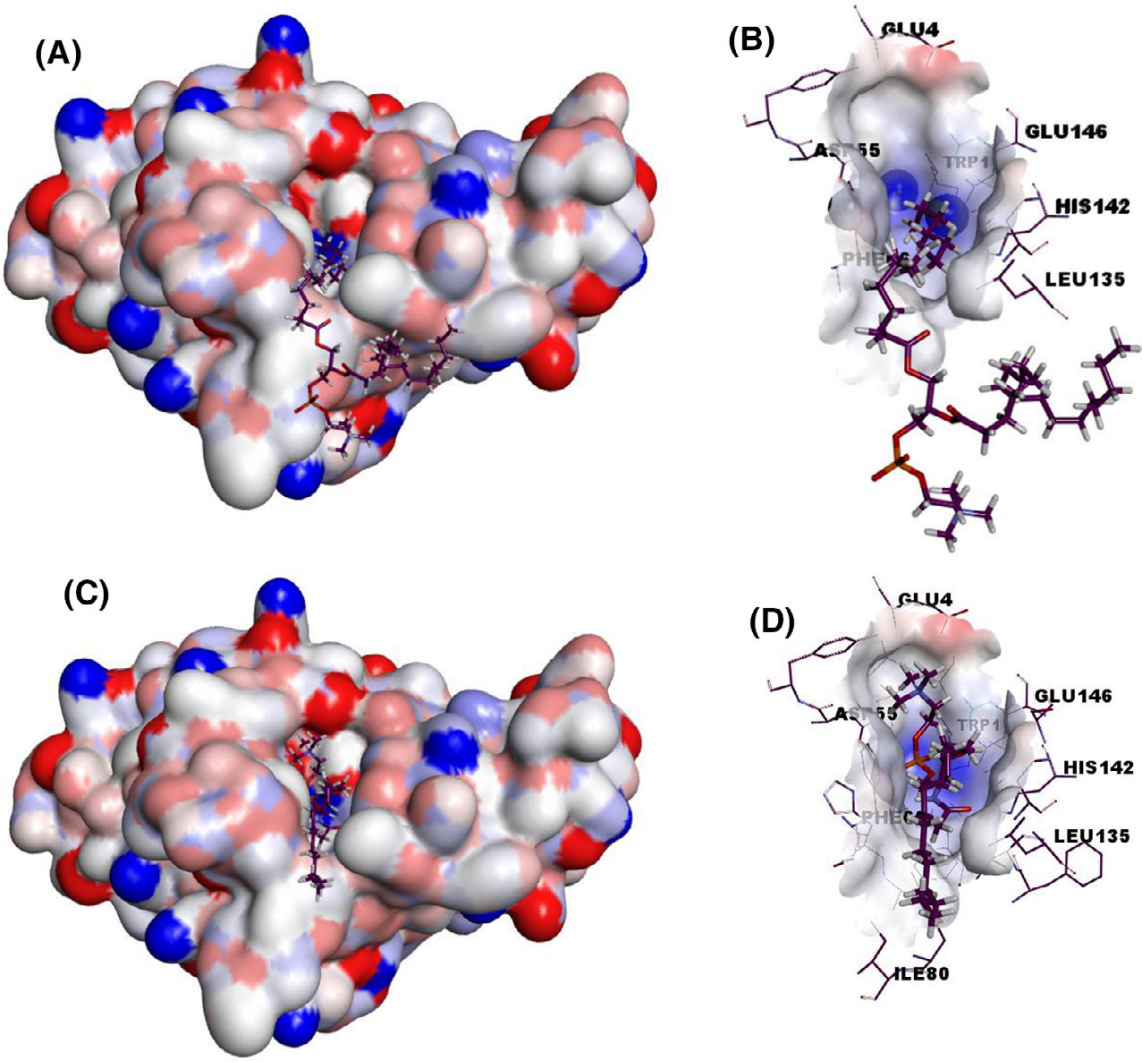
The electrostatic potential surface of LmPlcB with docked PC and SM. The generated model of LmPlcB (Fig. 1A) was used as a target for docking studies. SwissDock web service (http://www.swissdock.ch/docking) was used to predict the molecular interactions that may occur with different phospholipids: PC (PubChem CID:5497103) (A and B) and SM (ZINC56870813) (C and D). The docked structures were visualized using Discovery Studio Visualizer.

Docking studies with PC and SM were used for visualizing their interaction with the LmPlcB catalytically active site (Fig. 2).

LmPlcB is catalytically active at a wide pH range (5.0–8.0) [13, 32], although the recombinant protein exhibits optimal activity at acidic pH [14]. During cell‐to‐cell spread of Listeria, a decrease in pH triggers the metalloprotease Mpl, known to activate pro‐LmPlcB [42]. This pH change increases the active mature form of LmPlcB, which helps bacteria escape from the double‐membrane vacuole [42]. One mechanism by which pH regulates Mpl protease activity is by influencing the charge of a His residue that acts as a pH sensor [16]. Activation of pro‐LmPlcB is also mediated by an Mpl‐independent pathway, utilizing host cysteine proteases [43]. Finally, LmPlcB catalytic activity is regulated by vacuolar pH, and its compartmentalization to the spreading vacuole is critical for intracellular survival in neutrophils [44].

CpPLC hydrolyzes PC and SM [45, 46, 47, 48], with phosphatidylcholinesterase activity being five‐fold higher than sphingomyelinase C activity [49]. This enzyme also hydrolyzes PE, PI, PS, and PG [50]. This broader substrate specificity might allow *C. perfringens* to infect different tissues and help the bacterium escape the early phagosome toward the macrophage cytoplasm [50, 51]. Using Langmuir monolayer, vesicles, and red blood cells, it has been shown that CpPLC acts in a two‐stage process with an initial, rapid hydrolysis stage followed by membrane insertion when the hydrolysis products change the membrane dipole potential [52].

**Fig. 3:**
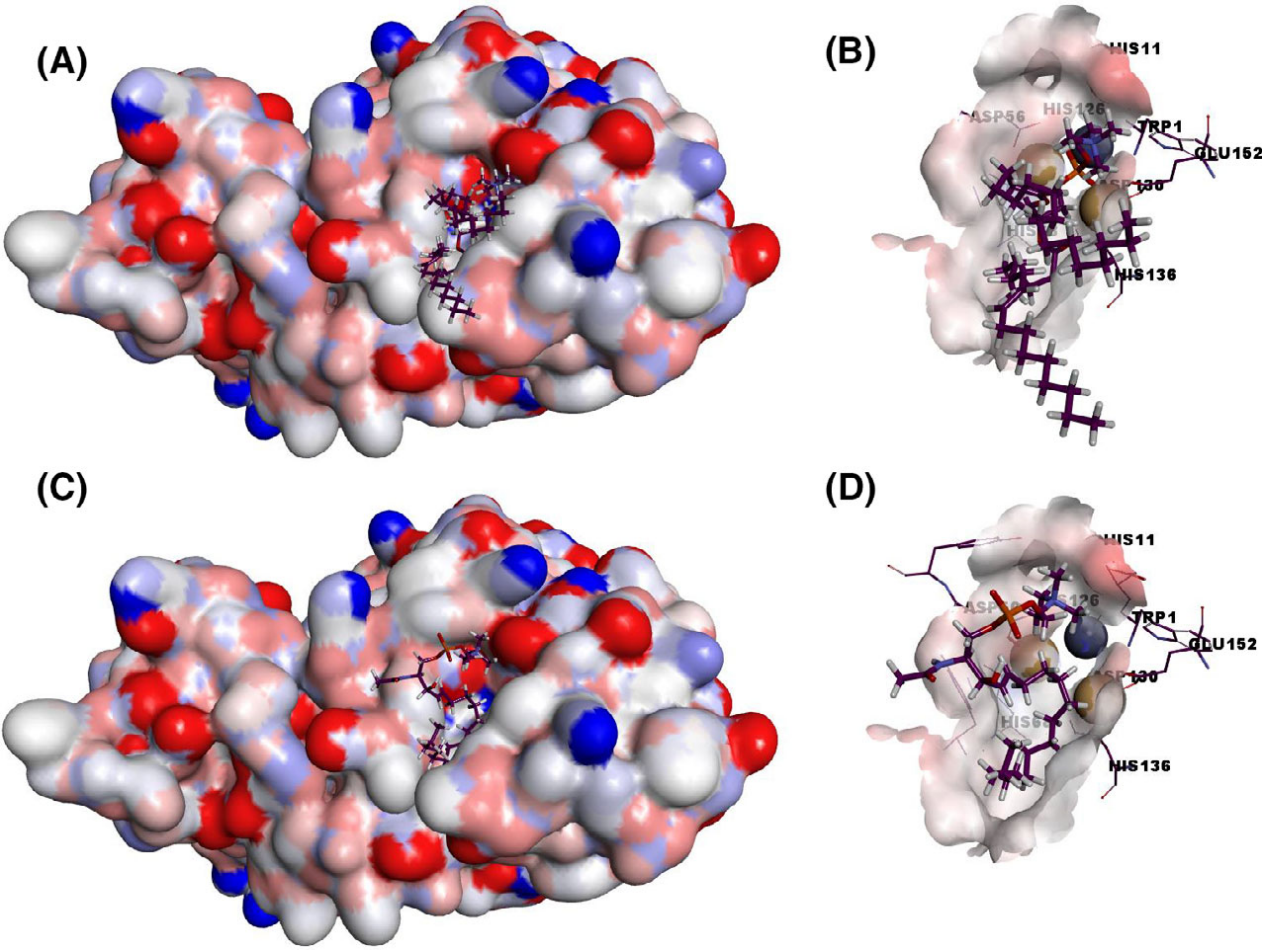
The electrostatic potential surface of CpPLC with docked PC and SM. The structure of CpPLC (PDB accession no. 1ca1) was used as a target for docking studies. SwissDock web service (http://www.swissdock.ch/docking) was used to predict the molecular interactions that may occur with PC (PubChem CID:5497103) (A and B) and SM (ZINC56870813) (C and D). The docked structures were visualized using the Discovery Studio Visualizer.

Docking studies with PC and SM were used for visualizing their interaction with the CpPLC catalytically active site (Fig. 3).

PaPlcH hydrolyzes mainly PC and SM, but it also acts on cardiolipin, PE, and PG when assayed on large unilamellar vesicles [53, 54]. However, the catalytic mechanism of PaPlcH is different from that of the zinc‐metalloPLCs. PaPlcH enzymatic activity is not affected by cation chelators [EDTA and egtazic acid (EGTA)], and instead of requiring zinc for activity, it is inhibited by Zn^+2^ and several divalent cations [27]. Furthermore, PaPlcH is not affected by the zinc‐metalloPLCs inhibitor D609 [27].

**Fig. 4:**
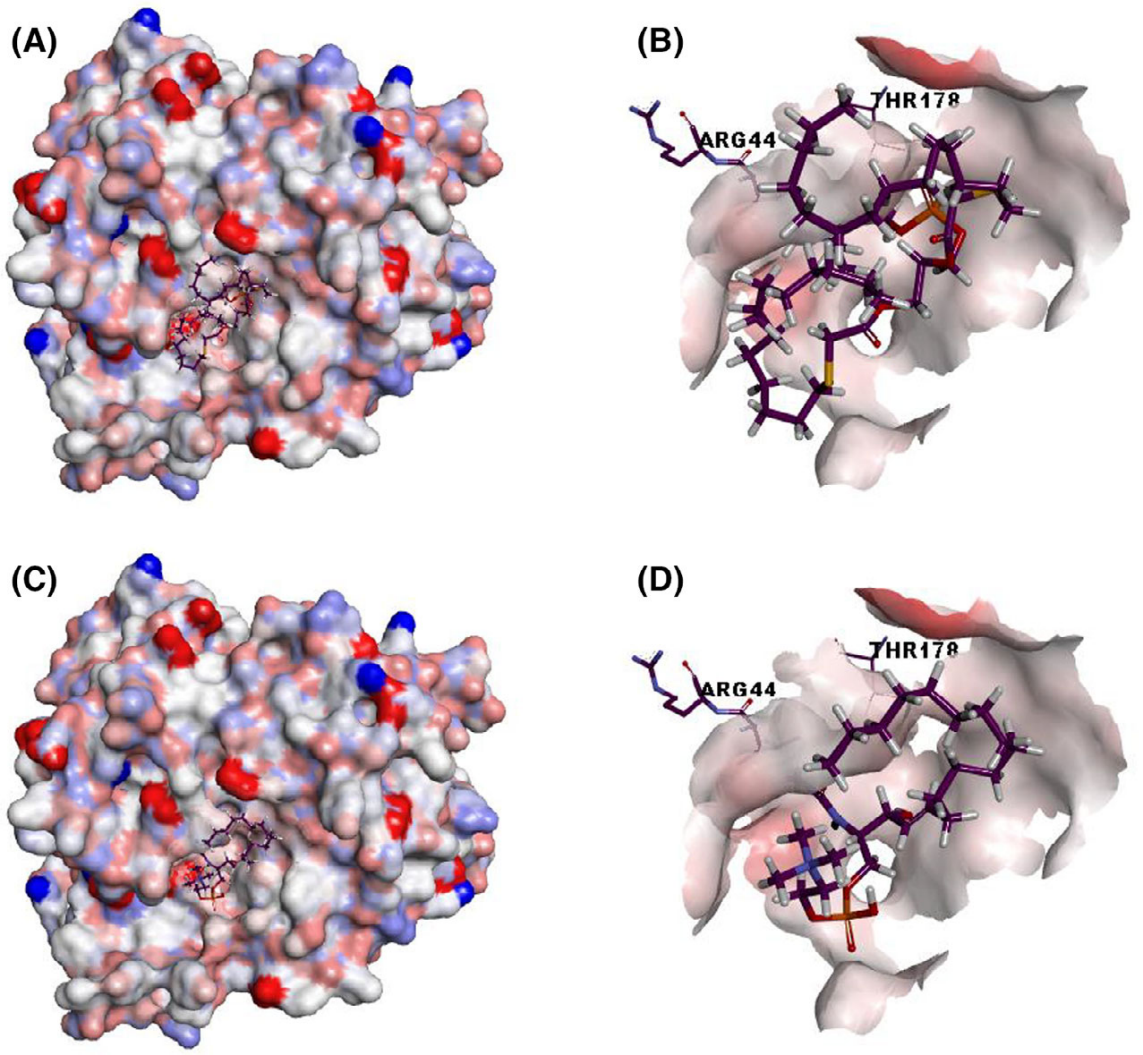
The electrostatic potential surface of PaPlcH with docked PC and SM. The generated model of PaPlcH (Fig. 1C) was used as a target for docking studies. SwissDock web service (http://www.swissdock.ch/docking) was used to predict the molecular interactions that may occur with PC (PubChem CID:5497103) (A and B) and SM (ZINC56870813) (C and D). The docked structures were visualized using Discovery Studio Visualizer.

Figure 4 shows docking studies of PaPlcH with PC and SM. The results show that both ligands bind to a similar location of the phosphate‐interacting and active sites residues of the ApcA structure, including the putative PaPlcH nucleophile Thr178.

PaPlcH partially purified from a clinical isolate showed enzymatic activity from pH 5.5–8, optimal at 7–8 [54]. However, studies on the effect of pH on the enzymatic activity with recombinant PlcH remain to be done.

The substrates for the bacterial enzymes discussed in this review and their corresponding optimal pH are summarized in Table 1.

**Table 1:** Broad substrate specificities and optimal pH from LmPlcB, CpPLC, and PaPlcH.

Enzyme	Substrates	pH activity	References
L. monocytogenes PlcB	PC, SM, PE, PG, PI, Cardiolipin, Plasmalogens, Plasmenylethanolamine (Glycerol acetal), PS	5.0–8.0 optimal: 5.0–6.0	[13, 14, 32, 33]
*C. perfringens* PLC	PC, SM, PE, PG, PI, PS	4.5–7.5 PC: optimal 5.0–6.0 SM: optimal 7.0	[45, 47, 49, 50]
*P. aeruginosa* PlcH	PC, SM, PE, PG, Cardiolipin, Plasmalogens, Lyso‐PC	5.5–8.0 Optimal 7.0–8.0	[27, 53, 54]

## Mechanisms of cytotoxicity

### Effects on erythrocytes

Iron is an essential resource for bacteria, and in vertebrate hosts, high amounts of iron are incorporated into hemoglobin [2]. Thus, accessing the iron stored in erythrocytes may constitute a significant advantage for bacterial growth during infections [2]. The three described Phospholipases C (PLCs) are hemolytic to a certain degree, and other effects on red blood cells are just starting to be elucidated, as described below.

LmPlcB is hemolytic, and this activity requires the presence of PC in the plasma membrane of the target erythrocytes [55]. Erythrocytes containing PC in their membranes, such as those of guinea pig, horse, and human, are hemolyzed by the enzyme. In contrast, sheep erythrocytes, which almost lack PC in their membrane, are resistant to hemolysis [13, 14].

Initial studies with *C. perfringens* supernatants evidenced their hemolytic activity when injected into pigeons, sheep, and rabbits [56]. This activity was optimal at 37 °C on the rabbit and human erythrocytes, whereas the hemolysis increased after cooling down the preparations to 4 °C in sheep erythrocytes [56, 57]. Further studies with highly purified CpPLC confirmed its hot‐cold hemolytic activity toward sheep erythrocytes [58, 59, 60]. CpPLC hemolyses horse erythrocytes by hydrolyzing mainly PC and sheep erythrocytes by hydrolyzing SM [57, 61]. Later studies using recombinant CpPLC showed that it causes hot‐cold hemolysis in red blood cells from different species [62, 63, 64, 65, 66]. Site‐directed mutants of CpPLC affecting either the Ca^+2^ or the Zn^+2^ binding domain, which affect catalytic activity against aggregated substrates, lack hemolytic activity [22, 62, 63, 64]. CpPLC lacking the linker between the C‐ and the N‐Terminal domains does not induce hemolysis of murine red blood cells, demonstrating that the interaction of both domains is necessary for its hemolytic activity [64, 65, 66].

A two‐stage interaction of CpPLC and human erythrocytes was shown using thermal shape fluctuation spectroscopy [52]. This finding supports a model in which the toxin is first incorporated into the membrane and second exerts its hydrolytic action [52]. At low concentrations, CpPLC induces morphological changes and a decrease of the membrane dipole potential that affects the erythrocyte membrane function [52]. At higher CpPLC concentrations, the morphological changes are accompanied by hemoglobin loss [52]. Additionally, CpPLC impairs erythropoiesis by inhibiting erythroid differentiation in mouse bone marrow cells [67].

PaPlcH caused hot‐cold hemolysis of human and sheep erythrocytes [27]. The hot‐cold hemolysis induced by this enzyme also occurs on goat erythrocytes lacking PC, but not on horse erythrocytes with PC but almost no SM. It suggests that SMase activity is critical for inducing hot‐cold hemolysis [68]. Accordingly, a neutral ceramidase encoded by a gene adjacent to PaPlcH enhances its hemolytic activity [69].

### Effects on platelets

CpPLC causes platelet aggregation *in vitro* and *in vivo* [24, 70, 71, 72]. This aggregation is induced by the translocation of the fibrinogen receptor gpIIbIIIa from internal stores to the membrane [73]. The effect of CpPLC on gpIIbIIIa involves intracellular calcium increments mediated by plasma membrane calcium channels [74]. Similarly, PaPlcH causes human platelet aggregation, which is dependent on its enzymatic activity [75].

### Effects on nucleated cells

The cellular effects of PLCs with phosphatidylcholinesterase and SMase activities have been evaluated on different cell lines, as discussed below.

A double mutant of both the LmPlcB and the PI‐PLC genes shows severely diminished cell‐to‐cell spread effect in a plaque assay with L2 fibroblasts [5]. Accordingly, an LmPlcB mutant induces fewer zones of dead cells when compared to the wild‐type bacteria [39]. J744 macrophage monolayers treated with an LmPlcB mutant of *L. monocytogenes* observed under electron microscopy showed significantly less lysis of the two‐membrane vacuoles that surround the bacteria after cell‐to‐cell spread than the wild‐type [39]. Infection studies on J744 macrophages have also helped to elucidate the proteolytic pathways that lead to activation of LmPlcB [43].

In murine bone marrow‐derived macrophages, both PI‐PLC and LmPlcB facilitate membrane disruption by acting on the inner membrane of the spreading vacuole, whereas LLO is required to degrade the outer membrane [40]. Studies with *L. monocytogenes* mutants lacking LLO and LmPlcB or Mpl showed that the last two are required for bacterial growth and lysis of the primary vacuole in epithelial cells, suggesting that the relative importance of LLO, the phospholipases, and Mpl varies in different cell types [34]. Accordingly, LLO is not required for lysis of *L.fnd="ER"> monocytogenes*‐containing primary vacuoles in some epithelial cell lines, such as Hep‐2 and HeLa [35, 36] or dendritic cells [37]. LmPlcB is specifically required for lysis of the primary vacuole of Hep‐2 and Hela cells in the absence of LLO [38]. An LLO negative‐LmPlcB inducible double mutant was also unable to establish cell‐to‐cell spread on Henle 407 cells without continuous LmPlcB induction during infection [38]. In a plaque assay created to visualize cell‐to‐cell spread from human macrophage U937 toward human epithelial Hep‐2 cells, it was determined that both PI‐PLC and LmPlcB are required to mediate escape from heterologous double‐membrane spreading vacuoles in the absence of LLO expression [40]. Furthermore, LmPlcB and PI‐PLC are critical for increasing ceramide, activating NFkB, and up‐regulating E‐selectin, as required for neutrophil adhesion to endothelial cells [76, 77].

The first report of the action of CpPLC on nucleated cells was to determine the membrane damage effect on human diploid fibroblasts and Hela cells [78]. By labeling these cells with 3^H^‐uridine and detecting the leakage of radioactive compounds, CpPLC treatment for 30 min was found to result in 25% of radioactivity released at concentrations of 16 µg·mL^−1^, reaching 100% maximum release after 1 h associated with the hydrolysis of membrane phospholipids [78]. Sarcoma 180 cells showed increased sensitivity toward CpPLC, whereas L1210 leukemia cells were relatively resistant to the cytotoxic effects of this enzyme [79]. CpPLC‐treated sarcoma cells also exhibited PC hydrolysis, alterations in size and shape, microvilli loss, and fistulas in cell membranes [79]. Later, a newly isolated fibroblast mutant, Don Q, had higher sensitivity to CpPLC than the wild‐type [80] due to a single‐point mutation in the UDP‐glucose pyrophosphorylase gene leading to UDP‐glucose deficiency [81, 82]. Since UDP‐glucose is a precursor of ganglioside synthesis, this mutation reduces cellular gangliosides in the outer cell membrane, which explains the increased susceptibility of Don Q to CpPLC. The gangliosides present in the cell membrane probably exert a steric effect or induce an electrostatic charge preventing the toxin from reaching its substrate [83]. Muscle is known to have the lowest concentration of complex gangliosides among all mammalian tissues [84, 85]. This may explain why muscle fibers present higher susceptibility to the cytotoxic effect of CpPLC during gas gangrene [83]. This hypersensitive cell line has been used to evaluate the cytotoxic activity of different CpPLC mutants and as a model to elucidate the mechanism of action of this toxin [62, 64, 83, 86, 87].

Several other cell types, such as cultured intestinal epithelial cells, Chinese hamster ovary (CHO), and Hela cells, have been used to study the effects of DAG generation induced by CpPLC, such as arachidonic acid release, PKC, PLA2, and calcium activation [88, 89, 90, 91, 92]. CpPLC action on endothelial cells showed DAG‐mediated induction of prostacyclin and platelet‐activating factor and increased adhesion molecules, both in endothelial cells and neutrophils, contributing to the process of vascular permeability and neutrophil recruitment [93]. Further studies in neutrophils showed that CpPLC induces respiratory burst through different signaling pathways, involving Tyr kinase A receptor (TrkA) and G proteins, leading to PKC theta and MEK‐ERK activation [94, 95]. Accordingly, CpPLC induces reactive oxygen species (ROS) production in hypersensitive cells through PKC or MEK‐ERK‐NFkB pathways that play an essential role in its cytotoxic/myotoxic effect [87, 96]. Epithelial cells have been used to demonstrate that CpPLC, TrkA, and GM1a ganglioside clustering activates the p38 MAPK pathway and PLC‐gamma1, ERK‐NFkB pathways to generate TNF‐alpha and IL‐8, important mediators of inflammation [97, 98, 99]. CpPLC induces the expression of granulocyte colony‐stimulating factor in human umbilical vein endothelial (HUVEC) cells via JNK activation [100]. Further studies showed that CpPLC causes apoptosis of endothelial cells by increasing ceramide [101]. Interestingly, the myoblast C2C12 cells are more resistant to ceramide‐induced cell death than HUVEC cells, suggesting that endothelial damage could be a primary effect caused by CpPLC [101].

PaPlcH induces the release of prostaglandins and leukotrienes in human granulocytes and mouse peritoneal cells [102]. PaPlcH is cytotoxic to mouse peritoneal macrophages and human mononuclear leukocytes and neutrophils [103]. Purified PaPlcH at low concentrations induces a dose‐dependent increase in IL‐8 expression in human isolated monocytes, which could be important during pathogenesis because IL‐8 is probably the main cause of the excessive neutrophil recruitment associated with pulmonary inflammation and destruction [104]. A PaPlcH mutant was significantly less cytotoxic to a human monocytic cell line (THP‐1) and a human T‐cell line (Jurkat) than the wild‐type strain, having a synergistic effect with the *P. aeruginosa* extracellular protease LepA [105].

PaPlcH suppresses respiratory burst in neutrophils by interfering with a PKC‐dependent pathway [106]. Thus, a bacterial strain mutant containing a targeted deletion of the plcHR operon induces a more robust oxidative burst than the wild‐type [106]. Furthermore, the addition of purified PaPlcH to the DeltaHR‐stimulated neutrophils suppresses superoxide production induced by phorbol esters to levels stimulated by wild‐type bacteria [106].

PaPlcHR is slightly cytotoxic to lung epithelial cells from a patient with cystic fibrosis and to HeLa cells, and highly toxic (at picomolar concentrations) to CHO and HUVEC [107]. PaPlcHR induces calcium signaling and caspase 3 activation on endothelial cells, and the selectivity toward endothelial cells is mediated by an RGD motif, probably by binding a subset of integrins [107]. The toxin also inhibits migration, invasion, and tubule formation by HUVEC, which points toward an antiangiogenic effect of PaPlcH [107].

## Effects on animal models of infection

Studying the effects of bacterial PLCs with phosphatidylcholinesterase and SMase activities or bacterial mutant strains lacking their corresponding genes in animal models has revealed important insights of these enzymes in virulence.

While LmPlcB is not toxic even when up to 25 µg is injected intravenously into mice [13], a *L. monocytogenes* mutant strain lacking LmPlcB is 20‐fold less lethal to mice [5]. Mice infected with the LmPlcB mutant survived cerebral listeriosis longer and have a reduced intracerebral bacterial load than mice infected with the wild‐type strain [108]. An *mpl* mutant that fails to produce mature LmPlcB showed impaired virulence in mice compared with a wild‐type strain and, at later stages of infection, exhibits less growth in mouse liver and spleen [109]. It has been shown that LmPlcB activation by Mpl inside vacuoles is important for its virulence because an LmPlcB pro‐mutant, secreted as an already mature and active enzyme, is strongly attenuated in a mouse infection model [110]. Later, it was found that the LmPlcB pro‐mutant interferes less with mitochondrial homeostasis than wild‐type strain, and is more susceptible to intracellular killing by peritoneal mice neutrophils, which explains its impaired virulence [44].

CpPLC is the main virulence factor in gas gangrene [7, 10]. Intramuscular injection of recombinant CpPLC causes myonecrosis and histopathological features of gas gangrene [93]. Immunization with the recombinant C‐terminal domain of this toxin protects mice from the intramuscular challenge of *C. perfringens* [111]. A *C. perfringens plc* mutant cannot induce gas gangrene in mice [10, 112]. CpPLC induces myonecrosis, leucocyte accumulation, thrombosis, and neutrophil arrestment in experimental gas gangrene [10, 112]. CpPLC variants, lacking the C‐terminal domain, mutated in residues of Ca^+2^ binding, or lacking surface‐exposed hydrophobic residues, failed to produce myonecrosis in a gas gangrene murine model [48, 62, 64, 113]. The importance of inflammatory mediators, such as TNF‐alpha, and the role of ROS production in CpPLC toxic effect has been demonstrated *in vivo* [96, 114]. Studies indicated that granulocyte colony‐stimulating factor does not reduce CpPLC induced myonecrosis in mice [100]. On the other hand, CpPLC decreases endothelial cell counts in mouse muscles infected with *C. perfringens*, which could contribute to myonecrosis progression [101].

PaPlcH causes dermonecrosis, vascular permeability, and death when injected intraperitoneally into mice [115]. This enzyme induces an inflammatory response characterized by the accumulation of inflammatory cells and the release of arachidonic acid metabolites [102]. PaPlcH induces recruitment and activation of platelets at the endothelium, leading to thrombotic lesions similar to those observed in *P. aeruginosa* sepsis [107]. PaPlcH in the respiratory tract induces inflammation, high levels of tumor necrosis factor‐alpha, interleukin 1‐beta, IL‐6, gamma interferon, MIP‐1 alpha, and MIP‐2 in the lungs [116]. A *P. aeruginosa* strain PaPlcH mutant exhibits decreased virulence in a murine burn infection model [117]. Similarly, in a mouse model of acute systemic infection by *P. aeruginosa*, a PaPlcH mutant showed reduced virulence compared to the wild‐type strain [105]. The numbers of mutant bacteria recovered in peritoneal lavage fluid and blood are lower than the numbers of the wild‐type strain [105].

Phosphorylcholine release from the pulmonary surfactant lipids by PlcH during lung infections provides nutrients to *P. aeruginosa*, and activates biofilm formation and anaerobic metabolism [118, 119, 120]. Furthermore, Cer accumulation can contribute to the pathogenesis of *P. aeruginosa* lung infections by inhibiting the function of the cystic fibrosis transmembrane conductance regulator Cl channel in epithelial cells, leading to thick mucus production that clogs the airways, which fosters bacterial growth [121].

## Concluding remarks

Bacterial PLCs with phosphatidylcholinesterase and sphingomyelinase C activities, such as LmPlcB, CpPLC, and PaPlcH, can exert diverse toxic effects. As observed in *in silico* docking assays while acting over different substrates, they generate 1,2‐diacylglycerol and ceramide, which change the biophysical properties of cellular target membranes. These products can undergo spontaneous transbilayer movement and recruit signaling proteins, affecting cellular processes such as cell cycle arrest, stress responses, autophagy, apoptosis, and cytokine production. The particular role of the bacterial enzymes in pathogenesis depends on the context of the infection. LmPlcB is activated when *L. monocytogenes* reaches the acidic environment of late endosomes‐lysosomes, aiding bacterial escape toward the cytosol and cell‐to‐cell propagation. On the other hand, when *C. perfringens* reaches a certain density in soft tissues and CpPLC is produced, this toxin induces endothelial damage and platelet aggregation, which favors ischemia, thus providing an optimal environment for bacterial growth. Finally, PaPlcH secreted by *P. aeruginosa*, hydrolyzes target phospholipids where the infection is being established, helps bacteria to obtain substrates from the host, and triggers signaling pathways that lead to inflammation. Several other bacteria such as *Burkholderia pseudomallei* and *Mycobacterium tuberculosis* produce enzymes homologous to PaPlcH, but their role in pathogenesis remains to be clarified. The data already available for LmPlcB, CpPLC, and PaPlcH can provide insights to further our understanding of their mechanism of action.

## Conflict of interest

The authors declare no conflict of interest.

## Author contributions

LM‐G wrote the first draft of the manuscript; FV‐R did the docking studies; MF‐D and AA‐G review and substantively edited the manuscript. All authors read and approved the final version and take responsibility for the overall content and integrity of the work.

## Data Availability

The data that support the findings of this study are available on request from the corresponding author.
